# Fibroblast growth factor receptor 3 protein is overexpressed in oral and oropharyngeal squamous cell carcinoma

**DOI:** 10.1002/cam4.595

**Published:** 2015-12-28

**Authors:** Koos Koole, Pauline M. W. van Kempen, Justin E. Swartz, Ton Peeters, Paul J. van Diest, Ron Koole, Robert J. J. van Es, Stefan M. Willems

**Affiliations:** ^1^ Department of PathologyUniversity Medical Center UtrechtHeidelberglaan 1003584 CXUtrechtThe Netherlands; ^2^ Department of Head and Neck Surgical OncologyUMC Utrecht Cancer CenterUniversity Medical Center UtrechtHeidelberglaan 1003584 CXUtrechtThe Netherlands; ^3^ Department of Otorhinolaryngology – Head and Neck SurgeryUniversity Medical Center UtrechtHeidelberglaan 1003584 CXUtrechtThe Netherlands; ^4^ Department of Molecular CarcinogenesisNetherlands Cancer InstitutePlesmanlaan 1211066 CXAmsterdamThe Netherlands

**Keywords:** fibroblast growth factor receptor 3, oral cancer, oropharyngeal cancer, therapeutic target

## Abstract

Fibroblast growth factor receptor 3 (FGFR3) is a member of the fibroblast growth factor receptor tyrosine kinase family. It has been identified as a promising therapeutic target in multiple types of cancer. We have investigated FGFR3 protein expression and *FGFR3* gene copy‐numbers in a single well‐documented cohort of oral and oropharyngeal squamous cell carcinoma. Tissue microarray sets containing 452 formalin‐fixed paraffin‐embedded tissues were immunohistochemically stained with an anti‐FGFR3 antibody and hybridized with a *FGFR3* fluorescence in situ hybridization probe. FGFR3 protein expression was correlated with clinicopathological and survival data, which were retrieved from electronic medical records. FGFR3 mRNA data of 522 head and neck squamous cell carcinoma (HNSCC) were retrieved from The Cancer Genome Atlas (TCGA). Fibroblast growth factor receptor 3 (FGFR3) protein was overexpressed in 48% (89/185) of oral and 59% (124/211) of oropharyngeal squamous cell carcinoma. Overexpression of FGFR3 protein was not related to overall survival or disease‐free survival in oral (HR[hazard ratio]: 0.94; 95% CI: 0.64–1.39; *P* = 0.77, HR: 0.94; 95% CI: 0.65–1.36; *P* = 0.75) and oropharyngeal squamous cell carcinoma (HR: 1.21; 95% CI: 0.81–1.80; *P* = 0.36, HR: 0.42; 95% CI: 0.79–1.77; *P* = 0.42). FGFR3 mRNA was upregulated in 3% (18/522) of HNSCC from the TCGA. The *FGFR3* gene was gained in 0.6% (1/179) of oral squamous cell carcinoma but no amplification was found in oral and oropharyngeal squamous cell carcinoma. In conclusion, FGFR3 protein is frequently overexpressed in oral and oropharyngeal squamous cell carcinoma. Therefore, it may serve as a potential therapeutic target for FGFR3‐directed therapies in oral and oropharyngeal squamous cell carcinoma.

## Introduction

Fibroblast growth factor receptor 3 (FGFR3) is a cell membrane‐bound tyrosine kinase receptor belonging to the fibroblast growth factor receptor family (FGFR1‐4) 1. Upon binding of specific FGF ligands, the receptor is phosphorylated and multiple downstream signaling pathways are activated. Among these pathways are the mitogen‐activated protein kinase (MAPK), (phosphoinositide 3‐kinase/protein kinase B (PI3K/AKT), phosphoinositide phospholipase C (PLC*γ*) and signal transducer and activator of transcription (STAT) signaling pathways 2. Activation of these pathways leads to cell proliferation, migration, invasion, cell survival, and angiogenesis. In cancer, oncogenic aberrations of the *FGFR3* gene cause sustained cell proliferation, contributing to tumor development 3. Genomic aberrations include *FGFR3* driver mutations, *FGFR3* gene amplification, and *FGFR3* translocations, which frequently occur in bladder cancer, myeloma, and glioblastoma 4, 5. Due to its contribution to tumor development, FGFR3 is an interesting therapeutic target and targeted therapies aimed at FGFR3 are emerging. Inhibiting FGFR3 protein with FGFR3‐directed therapies caused remarkable antitumor effects in preclinical models on brain cancer, colorectal cancer, and multiple myeloma, as well as in clinical trials on glioblastoma patients with tumors bearing *FGFR3‐TACC3* fusions 3, 4, 6. At the moment, early phase clinical trials are conducted with FGFR3‐directed targeted therapies on patients with *FGFR3*‐aberrated glioblastoma multiforme, transitional cell carcinoma, multiple myeloma, and other advanced solid malignancies (ClinicalTrials.gov Identifier: NCT01975701, NCT02278978, NCT02401542, NCT02052778).

For head and neck squamous cell carcinoma (HNSCC), the knowledge on FGFR3 protein expression is yet limited, while such novel therapeutic targets are highly awaited for disseminated or recurrent HNSCC because current treatment regimens are often ineffective and overall survival rates have remained poor over the past two decades 7, 8. In this study, we therefore investigated FGFR3 protein expression and its relation to overall survival, disease‐free survival, and regional lymph node metastases in well‐documented cohorts of oral and oropharyngeal squamous cell carcinoma (OSCC, OPSCC). Second, we investigated *FGFR3* gene copy‐numbers in these OSCC and OPSCC cohorts.

## Materials and Methods

### Patient cohort

The inclusion criteria were as follows: patients with a first primary OPSCC or OSCC who were treated at the University Medical Center Utrecht (UMC Utrecht) between August 1996 and December 2011. The exclusion criteria were as follows: a previous history of HNSCC, a synchronous primary tumor, histologic abnormalities including inflammation and dysplastic lesions, and the absence of tumor cores on tissue microarray (TMA) slides. The patient cohort included 212 OSCC and 240 OPSCC patients. TMA cores were absent for 27 OSCC and 29 OPSCC, leaving 185 OSCC and 211 OPSCC for analysis of FGFR3 protein expression. OSCC and OPSCC patients were treated according to the Dutch National Guideline for Head and Neck Cancer 2014 9. The treatment regimen of OSCC consisted of primary surgical resection and an additional neck dissection and/or postoperative radiotherapy or chemoradiotherapy if recommended. The treatment regimen of OPSCC consisted of primary surgery, radiotherapy, or chemoradiotherapy and an additional neck dissection or postoperative radiotherapy or chemoradiotherapy if recommended. For OSCC patients, the median follow‐up of overall survival was 90.5 months and of disease‐free survival was 91.5 months. For OPSCC patients, the median follow‐up time of overall survival was 71 months and of disease‐free survival was 63 months. Clinicopathological patient and tumor characteristics were retrieved from electronic medical records and formalin‐fixed paraffin‐embedded tissues of all tumors were collected. Oral squamous cell carcinoma (OSCC) tissues were surgical resection specimens as surgery was the standard treatment regimen for OSCC, and OPSCC tissues were mainly pretreatment biopsy specimens as chemoirradiation was the treatment regimen for the majority of OPSCC. Since limited OPSCC resection specimens were available for microscopic analysis by a pathologist, pathological characteristics of these tumors were not available. The Human Papillomavirus (HPV) status of all tumors was determined by P16 immunohistochemistry and linear array, as described previously 10. “The code for proper secondary use of human tissue” and “The code of conduct for the use of data in health research” of the Federation of Dutch Medical Scientific Societies were followed when handling human tissues and patient data (Federa FMVV, updated 2011).

### Tissue microarray construction

Slides (4 *μ*m) cut from formalin‐fixed paraffin‐embedded (FFPE) tissue blocks were hematoxylin stained and tumor areas were marked by a dedicated head and neck pathologist (SMW). Three 0.6 mm cores were punched from marked tumor areas of each FFPE tissue block and these were arrayed into recipient paraffin donor blocks using a TMA Grand Master (3DHISTECH, Budapest, Hungary). Six normal oral and tonsillar tissue TMA cores were arrayed in each TMA and served as staining quality controls.

### Immunohistochemistry

Tissue microarray (TMA) slides were immunohistochemically stained using a mouse monoclonal anti‐FGFR3 (B‐9) SC‐13121 antibody (Santa Cruz Biotechnology, Dallas, TX). The anti‐FGFR3 antibody was tested for the following tissue pretreatments; EDTA, citrate, pepsine and no treatment, and the following antibody dilutions; 1:10, 1:25, 1:50, 1:100, 1:250, 1:500, and 1:1000 on a positive control (normal liver tissue) and negative control (normal stomach tissue) to verify the antibody's specificity for FGFR3 protein 11. Tissue pretreatment with EDTA and a 1:25 antibody dilution showed the most specific staining. The following manual staining protocol was used; first, TMA slides were deparaffinized and treated with peroxidase inhibitor for 15 min, followed by an EDTA pretreatment step at 100°C for 20 min. After rinsing in demineralized water and PBS tween, slides were incubated with primary anti‐FGFR3 antibody at 1:25 dilution for one hour and rinsed in PBS sequenza. Next, slides were incubated with 150 *μ*L poly‐HRP‐anti‐mouse/rabbit/rat IgG secondary antibody (Immunologic, Duiven, The Netherlands) for 30 min, followed by rinsing in PBS sequenza and citrate buffer. 200 *μ*L 3,3’‐diaminobenzidine (DAB) was applied for 10 min and slides were rinsed in citrate buffer. Finally, slides were counterstained with hematoxylin, dehydrated, and covered.

To quantify FGFR3 protein expression, the resultant immunohistochemical stain was scored in a semiquantitative manner by two observers (S. M. W and K. K) blinded to patient's outcome. The percentage of positively stained tumor cells was scored and the mean tumor cell percentage was computed from available TMA cores for each tumor. Seventy‐four tumors included in the analysis had only one TMA core available and 138 tumors had only two. Continuous tumor cell percentage scores were dichotomized by a cut‐off value, which was optimized to best predicting patient overall survival using log‐likelihood values 12. A cut‐off value of 33% was selected for both OSCC and OPSCC. The staining intensity was not scored because it was homogenous among all TMA cores.

### Fluorescence in situ hybridization

Tissue microarrays were hybridized with an *IGH/FGFR3* (*IGH*, immunoglobulin heavy locus) translocation dual fusion FISH (fluorescence in situ hybridization) probe (Cytocell, Cambridge, UK). In brief, 4 *μ*m TMA slides were deparaffinized, rinsed in HCL solution, and pretreated with citrate and protease buffer. Next, these slides were dehydrated, and incubated with 15 *μ*L Fluorescence in situ hybridization (FISH) probe for 5 min at 78°C. After cooling samples for 5 min, TMA slides were incubated overnight at 37°C in a Thermobrite (Abbott Laboratories, Abbott Park, IL). The next day, TMA slides were rinsed in specific saline‐sodium citrate buffers and counterstained with 4’,6‐diamidino‐2‐phenylindole (DAPI). Finally, slides were dehydrated and 15 *μ*L vectashield was applied. To determine *FGFR3* gene copy‐numbers, 50 tumor cell nuclei per tumor were assessed on *FGFR3* and *IGH* gene copy‐numbers at 100× magnification using a Leica DM5500 B microscope system with Leica application suite advanced fluorescence software (Leica Microsystems, Rijswijk, The Netherlands). A *FGFR3/IGH* ratio was calculated and defined as: <1.5: normal copy‐numbers, 1.5–2.0: copy‐number gain and >2.0: gene amplification 13.

### TCGA data collection

FGFR3 mRNA data, available for 522 HNSCC, were retrieved from The Cancer Genome Atlas (TCGA) Research Network (http://cancergenome.nih.gov/). Data were extracted from the TCGA Head and Neck Squamous Cell Carcinoma Provisional study through the cBioPortal for Cancer Genomics website (http://www.cbioportal.org/) on the 29th of September, 2015 14, 15. Upregulated FGFR3 mRNA levels were defined as z‐scores above two standard deviations and downregulated FGFR3 mRNA levels as below two standard deviations from the reference population. No limitations or publication restrictions were laid upon the HNSCC data, as stated by the TCGA publication guidelines.

### Statistical analysis

Statistical analysis was performed using IBM SPSS Statistics software, version 22 (IBM, Amonk, NY). Pearson's chi square test was used for dichotomous variables or Fisher's exact test for the variables; primary treatment type, neck dissection and HPV status, and *t*‐test was used for continuous variables to compare baseline characteristics between OSCC and OPSCC. A Pearson's chi square test was used to compare protein expression between OSCC and OPSCC. A Pearson's chi square was also used to analyze univariate associations between FGFR3 protein expression and clinicopathological variables.

Only patients who were treated with curative intent were included in the survival analysis. The median follow‐up time was estimated using the reverse Kaplan–Meier method. The relation between FGFR3 protein expression and both overall survival and disease‐free survival was analyzed by plotting Kaplan–Meier survival curves and comparing them by log‐rank test. Associations were further analyzed by univariate Cox regression. Two‐sided *P*‐values below 0.05 were considered significant throughout all statistical computations.

## Results

### Clinicopathological characteristics

The OSCC patient cohort included 212 OSCC, of which 30% were early I–II stage tumors and 70% advanced III–IV stage tumors (Table 1). Regarding treatment regimens, all OSCC were primarily treated with surgery (100%) and 97% received a neck dissection. Postoperative radiotherapy was administered to the primary site and/or neck in 34%. The OPSCC patient cohort included 240 OPSCC, of which 15% were early I–II stage tumors and 85% advanced III–IV stage tumors. 19% of OPSCC were HPV‐positive and 81% HPV‐negative. HPV status was missing for nine OPSCC. Of all OPSCC's, 77% were treated either primarily or postoperative with chemo‐irradiation or radiotherapy. Twenty‐five percent were primarily treated with surgery. Eleven percent of OPSCC were treated with palliative intent.

**Table 1:** Clinicopathological characteristics of oral and oropharyngeal squamous cell carcinoma cohorts

Clinicopathological characteristics	OSCC *n* (%)	OPSCC *n* (%)	*P*
Total number of cases	212 (100)	240 (100)	
Age			
Median (range)	62 (26–87)	59 (35–88)	0.005
Sex			
Male	128 (60)	167 (70)	0.040
Female	84 (40)	73 (30)	
Tobacco smoking			
Never	77 (36)	48 (20)	0.012
Yes	133 (63)	192 (80)	
Missing	2 (1)	0 (0)	
Alcohol consumption			
Never	102 (48)	38 (15.5)	0.008
Yes	108 (51)	201 (84)	
Missing	2 (1)	1 (0.5)	
Clinical T‐stage			
cT1	44 (21)	21 (8.5)	<0.001
cT2	79 (37)	68 (28)	
cT3	19 (9)	56 (23)	
cT4	70 (33)	94 (39)	
Missing	0 (0)	1 (0.5)	
Clinical N‐stage			
cN0	146 (69)	60 (25)	<0.001
cN1‐3	66 (31)	175 (74)	
Missing	0 (0)	3 (1)	
Pathological N‐stage			
pN0	91 (43)	NA	NA
pN1‐3	114 (54)		
Missing	7 (3)		
Tumor stage			
Early I–II	64 (30)	36 (15)	<0.001
Advanced III–IV	148 (70)	204 (85)	
Primary treatment type			
Surgery	212 (100)	61 (25)	<0.001
Radiotherapy or chemoradiotherapy	0 (0)	153 (64)	
Palliative	0 (0)	26 (11)	
Neck dissection			
Yes	205 (97)	28 (12)	<0.001
No	7 (3)	186 (77)	
Palliative	0 (0)	26 (11)	
Postoperative Radiotherapy or chemoradiotherapy			
Yes	73 (34)	31 (13)	0.020
No	139 (66)	30 (13)	
Palliative	0 (0)	26 (11)	
Extra nodal growth			
No or pN0	154 (73)	NA	NA
Yes	56 (26)		
Missing	2 (1)		
Vaso‐invasion			
No	169 (80)	NA	NA
Yes	39 (18)		
Missing	4 (2)		
Perineural growth			
No	122 (58)	NA	NA
Yes	80 (38)		
Missing	10 (4)		
Bone invasion			
No	152 (72)	NA	NA
Yes	60 (28)		
Growth pattern			
Cohesive	44 (20.5)	NA	NA
Noncohesive	167 (79)		
Missing	1 (0.5)		
Infiltration depth			
0–4 mm	19 (9)	NA	NA
>4 mm	193 (91)		
Differentiation grade			
Well/moderate	173 (82)	NA	NA
Poor/undifferentiated	39 (18)		
HPV‐16			
Positive	2 (1)	43 (18)	<0.001
Negative	210 (99)	188 (78)	
Missing	0 (0)	9 (4)	

OSCC, oral squamous cell carcinoma; OPSCC, oropharyngeal squamous cell carcinoma.

### FGFR3 protein is frequently overexpressed in oral and oropharyngeal squamous cell carcinoma

**Figure 1:**
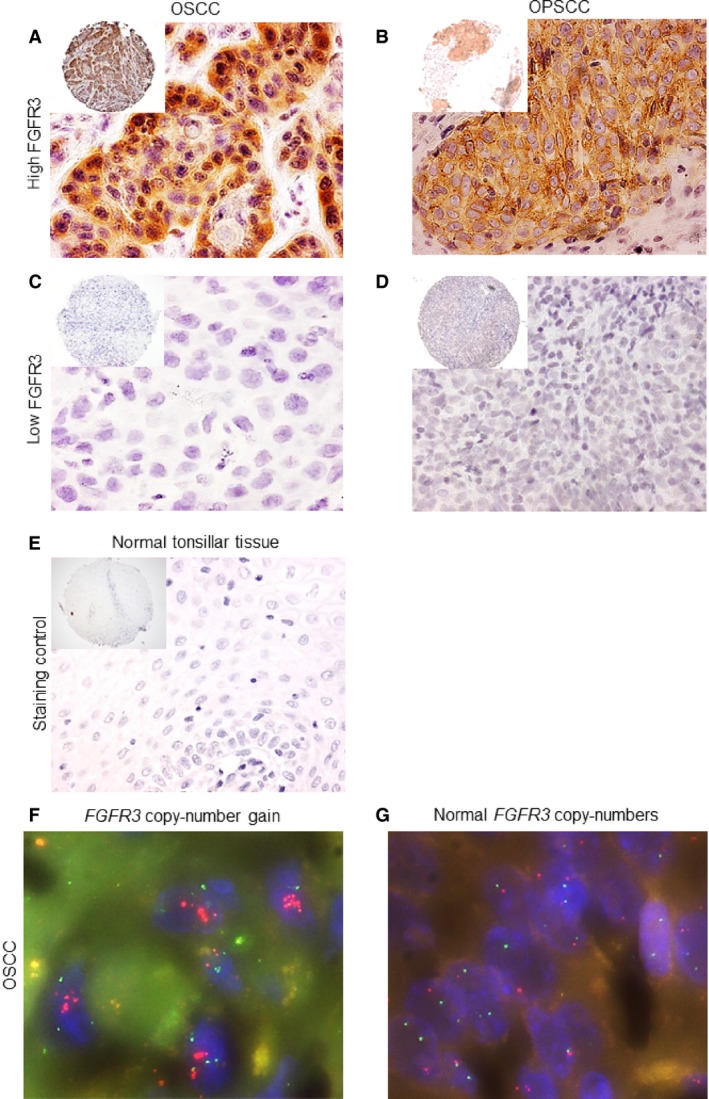
Microscopic images of immunohistochemical staining for FGFR3 protein at 10× and 40× magnification and fluorescence in situ hybridization of the *FGFR3* gene at 100× magnification. Tissue microarray slides containing oral and oropharyngeal squamous cell carcinoma cores (0.6 mm) were immunohistochemically stained for FGFR3 protein using an anti‐FGFR3 antibody. Fluorescence in situ hybridization of the *FGFR3* gene was performed on another set of tissue microarray slides. Strong immunohistochemical staining was observed in (A) oral squamous cell carcinoma and (B) oropharyngeal squamous cell carcinoma overexpressing FGFR3 protein. No staining was observed in (C) oral squamous cell carcinoma and (D) oropharyngeal squamous cell carcinoma not expressing FGFR3 protein. Normal tonsillar tissue (E) showed no staining for FGFR3 protein. (F) *FGFR3* copy‐number gain and (G) normal *FGFR3* gene copy‐numbers in oral squamous cell carcinoma. The red probe signal is hybridized to the *FGFR3* gene and the green probe signal is hybridized to the *IGH* gene. FGFR3, fibroblast growth factor receptor 3; OPSCC, oropharyngeal squamous cell carcinoma; OSCC, oral squamous cell carcinoma; *IGH*, immunoglobulin heavy locus.

**Figure 2:**
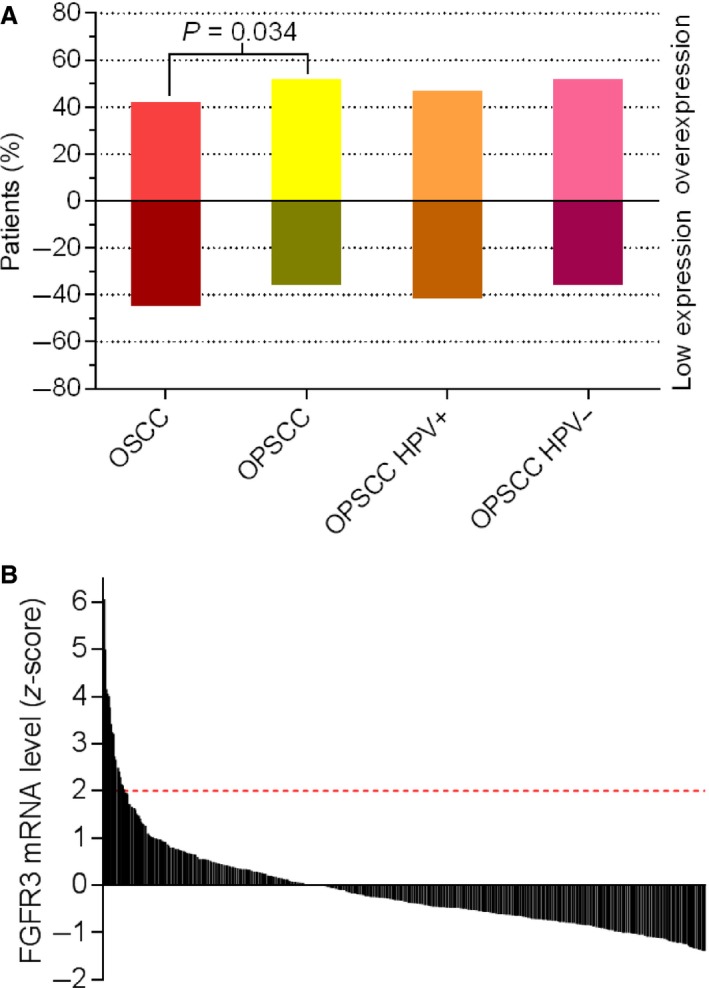
Prevalence of FGFR3 protein overexpression and FGFR3 mRNA levels in head and neck squamous cell carcinoma. (A) Tissue microarray slides containing oral and oropharyngeal squamous cell carcinoma cores (0.6 mm) were immunohistochemically stained for FGFR3 protein using an anti‐FGFR3 antibody. FGFR3 protein was overexpressed in 48% (89/185) of oral squamous cell carcinoma and 59% (124/211) of oropharyngeal squamous cell carcinoma. Within the oropharyngeal population, FGFR3 protein was overexpressed in 53% (20/38) of HPV‐positive and 59% (97/165) of HPV‐negative oropharyngeal squamous cell carcinoma. (B) Data on FGFR3 mRNA levels of 522 HNSCC were retrieved from The Cancer Genome Atlas (TCGA) Research Network on the 29th of September, 2015. FGFR3 mRNA levels were upregulated in 3% (18/522) of all HNSCC and normal in all other HNSCC head and neck squamous cell carcinoma. FGFR3, fibroblast growth factor receptor 3; HPV, human papillomavirus; OPSCC, oropharyngeal squamous cell carcinoma; OSCC, oral squamous cell carcinoma.

Representative microscopic images of FGFR3 protein expression are shown in Figure**** 1. A subset of OSCC and OPSCC showed more intense staining and a subset showed lighter staining compared to the faint staining observed in normal oral and tonsillar tissue. Specifically, FGFR3 protein was overexpressed in 48% (89/185) of OSCC and 59% (124/211) of OPSCC (Fig. 2A, Table 1). FGFR3 protein expression was unknown for 27 OSCC and 29 OPSCC because all three TMA cores of the tumor were missing. Overexpression of FGFR3 protein occurred significantly more in OPSCC (*P* = 0.034). Regarding the OPSCC population, there was no significant difference in FGFR3 protein expression between HPV‐positive and ‐negative OPSCC (*P* = 0.489). To investigate the underlying mechanism of FGFR3 protein overexpression in OSCC and OPSCC, FGFR3 mRNA data of 522 HNSCC were retrieved from The Cancer Genome Atlas (TCGA) Research Network. FGFR3 mRNA levels were upregulated in 3% (18/522) of HNSCC and normal in the rest of the HNSCC cohort (Fig. 2B). None of them showed downregulated FGFR3 mRNA levels. In the OSCC and OPSCC cohort, the *FGFR3* gene was gained in only 0.6% (1/179) of OSCC, but not truly amplified in OSCC nor OPSCC (Fig. 1, Table S1).

### FGFR3 protein expression is not related to overall survival or disease‐free survival in oral and oropharyngeal squamous cell carcinoma

**Figure 3:**
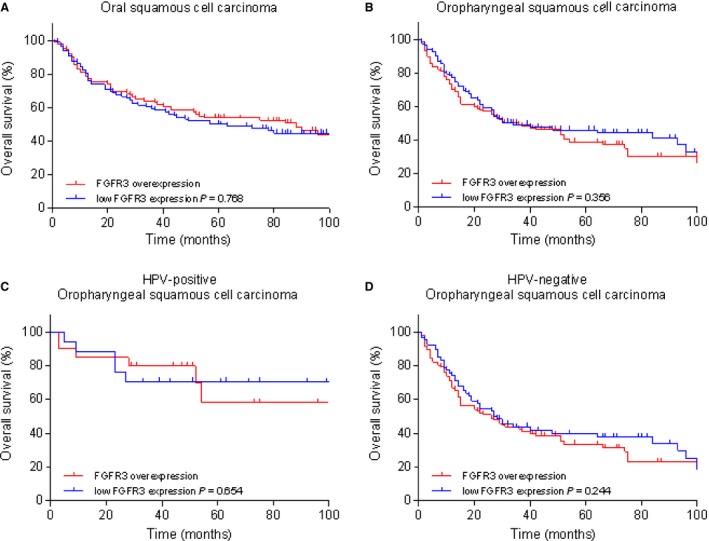
Kaplan–Meier overall survival curves for FGFR3 protein expression in oral and oropharyngeal squamous cell carcinoma. Tissue microarray slides containing cores (0.6 mm) of 212 oral and 240 oropharyngeal squamous cell carcinoma were immunohistochemically stained for FGFR3 protein using an anti‐FGFR3 antibody. FGFR3 protein expression was not related to overall survival in (**A**) oral squamous cell carcinoma (HR: 0.94; 95% CI: 0.64–1.39; *P* = 0.769) and (B) oropharyngeal squamous cell carcinoma (HR: 1.21; 95% CI: 0.81–1.80; *P* = 0.361). Similarly, FGFR3 protein expression was not related to overall survival in (C) HPV‐positive (HR: 0.74; 95% CI: 0.20–2.77; *P* = 0.657) and (D) HPV‐negative oropharyngeal squamous cell carcinoma (HR: 1.28; 95% CI: 0.84–1.97; *P* = 0.249). FGFR3, fibroblast growth factor receptor 3; HPV, human papillomavirus; HR, hazard ratio.

Expression of FGFR3 protein was not related to overall survival or disease‐free survival in OSCC patients (HR [hazard ratio]: 0.94; 95% CI: 0.64–1.39; *P* = 0.769, HR: 0.94; 95% CI: 0.65–1.36; *P* = 0.750). Also not in OPSCC patients (HR: 1.21; 95% CI: 0.81–1.80; *P* = 0.361, HR: 0.42; 95% CI: 0.79–1.77; *P* = 0.419) (Fig. 3, Fig. S1). Furthermore, FGFR3 protein expression was not related to overall survival or disease‐free survival in subgroups of HPV‐positive OPSCC (HR [hazard ratio]: 0.74; 95% CI: 0.20–2.77; *P* = 0.657, HR: 0.48; 95% CI: 0.14–1.64; *P* = 0.241) and HPV‐negative OPSCC patients (HR: 1.28; 95% CI: 0.84–1.97; *P* = 0.249, HR: 1.40; 95% CI: 0.90–2.16; *P* = 0.133). Fibroblast growth factor receptor 3 (FGFR3) protein expression showed no relevant relationship with clinicopathological variables, including differentiation grade, tumor stage, and regional lymph node metastases.

## Discussion

In this study, FGFR3 protein appeared to be frequently overexpressed in both OSCC and OPSCC, and FGFR3 mRNA was found to be upregulated in HNSCC from the TCGA Research Network. Upregulated FGFR3 mRNA levels may account for the overexpression of FGFR3 protein in a minor subset of FGFR3‐overexpressed OSCC and OPSCC samples. However, for the majority of FGFR3‐overexpressed samples, the mechanism of overexpression remains unknown. Similar overexpression of FGFR3 protein has been observed in a previous study. They observed FGFR3 protein overexpression in 13 of 14 OSCC cell lines and in five OSCC tissues 16. Their method was Quantitative Reverse Transcription Polymerase Chain Reaction (QRT‐PCR), whereas immunohistochemistry was used in this study. Since no consensus on a cut‐off value has been reached yet, we selected an arbitrary cut‐off value to define protein overexpression by immunohistochemistry. Fibroblast growth factor receptor 3 (FGFR3) protein has become an interesting therapeutic target as several FGFR inhibitor therapies have become available. These FGFR inhibitors show promising therapeutic value for treating HNSCC in in vitro and in vivo experiments. For example, targeting FGFR3 with FGFR‐inhibitor PD173074 reduced cell proliferation and enhanced radiotherapy sensitivity of resistant OSCC cell lines and xenografts 17. Hence, targeting FGFR3 could be of interest for radiotherapy treatment strategies in radiotherapy resistant OSCC patients.

Regarding other types of cancer, the overexpression of FGFR3 protein has been observed in a wide spectrum of solid tumors including transitional cell, hepatocellular, and breast carcinoma, as well as multiple myeloma 18, 19, 20, 21, 22, 23, 24. Similar overexpression of FGFR3 protein was observed in this study. Though, the contribution of FGFR3 protein overexpression to tumor progression seems to be tumor‐type dependent. FGFR3 protein overexpression drives tumor progression in bladder cancer, lung cancer, multiple myeloma, and glioblastoma, whereas it prevents tumor progression in cutaneous squamous cell carcinoma 25, 26. In HNSCC, FGFR3 protein overexpression seems to drive tumor progression, as reported by Uzawa et al. 17.

We found FGFR3 protein expression to be of no prognostic value in OSCC as well as in OPSCC. The prognostic value of FGFR3 protein expression has not been evaluated before 27. This holds both true for HPV‐negative and HPV‐positive tumors despite their different mechanism of FGFR3 activation. The prognostic value of FGFR3 protein expression seems to be tumor‐type dependent. Similar to this HNSCC study, FGFR3 protein expression holds no prognostic value in non‐small cell lung cancer 28. This is in contrast to multiple myeloma and breast cancer, in which FGFR3 protein expression has been related to poor progression‐free survival and overall survival 23, 24. For transitional cell carcinoma, the prognostic value for FGFR3 expression is still a subject of debate 18, 19, 29, 30, 31, 32, 33, 34.

Previous large‐scale genomic characterization studies show that the *FGFR3* gene is frequently aberrant in HPV‐positive HNSCC (11%) and much less in HPV‐negative HNSCC (2%) 35, 36. Specifically, *FGFR3‐TACC3* translocations occur predominantly in HPV‐positive HNSCC, whereas *FGFR3* amplification occurs in HPV‐negative HNSCC and *FGFR3* mutations occur in both. Tumors bearing these *FGFR3* gene aberrations respond very well to FGFR‐inhibitors in preclinical models and early phase clinical studies 37. Considering the frequent occurrence of *FGFR3* aberrations in HPV‐positive HNSCC and impressive response to FGFR‐inhibitors, FGFR3 protein might be of therapeutic value in this HNSCC subpopulation. Clinical studies are currently focusing on this HPV‐positive subpopulation 37. The *FGFR3* amplification observed by the previous study in a mixed HPV‐negative HNSCC cohort was not observed in the OSCC and OPSCC cohorts in this study 30. An explanation could be that the *FGFR3* gene is amplified in HNSCC tumors other than OSCC and OPSCC or gene amplification is not detected by our FISH analysis due to the arbitrarily selected ratio and cut‐off values. In the OSCC and OPSCC of this study, other mechanisms than *FGFR3* gene amplification are probably responsible for FGFR3 protein overexpression.

In conclusion, FGFR3 protein is frequently overexpressed in both OSCC and OPSCC. Although FGFR3 protein expression is not related to overall survival or disease‐free survival, previous studies found a high occurrence rate of *FGFR3* genomic aberrations in HNSCC. Therefore, FGFR3 protein may be an interesting therapeutic target for FGFR3‐directed therapies in OSCC and OPSCC.

## Conflict of Interest

All authors had no conflicts of interest to declare.

## Supporting information


**Figure S1.** Kaplan–Meier disease‐free survival curves for FGFR3 protein expression in oral and oropharyngeal squamous cell carcinoma.

**Table S1**. FGFR3 protein expression and *FGFR3* gene copy‐numbers in oral and oropharyngeal squamous cell carcinoma.
