# Supplementary figures and images for: Fibroblast growth factor receptor 3 protein is overexpressed in oral and oropharyngeal squamous cell carcinoma
Source: Cancer Med. 2015 Dec 28;5(2):275–84. doi: 10.1002/cam4.595 (PMC4735780; doi:10.1002/cam4.595)

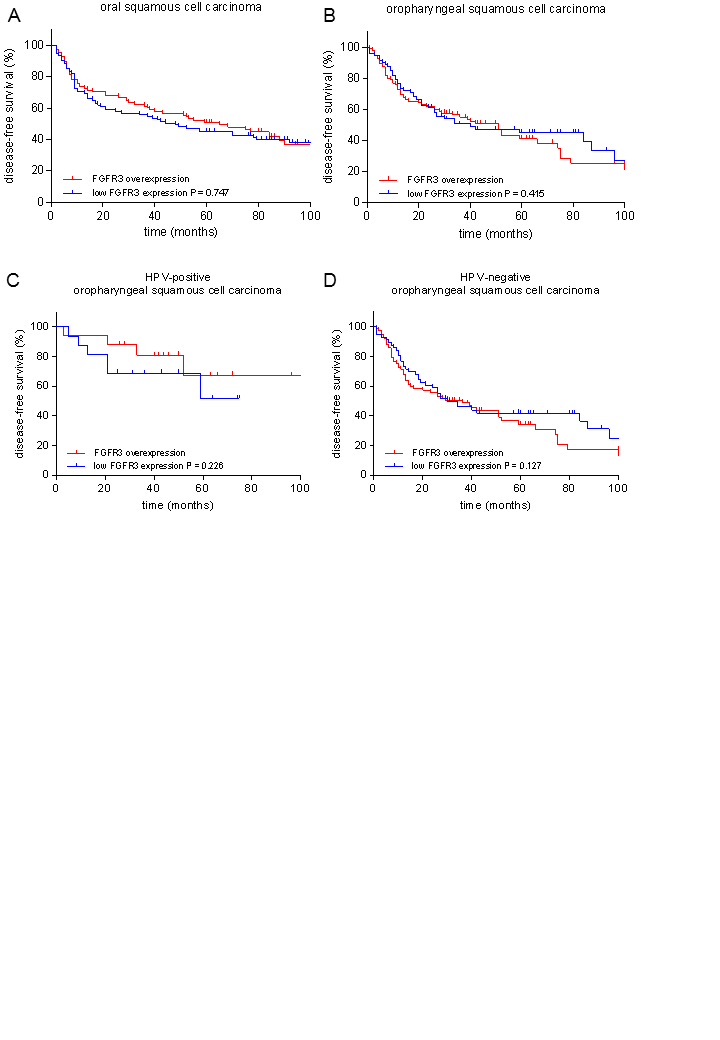

Supplement: Supplementary file 1 — Figure S1. Kaplan–Meier disease‐free survival curves for FGFR3 protein expression in oral and oropharyngeal squamous cell carcinoma. [file CAM4-5-275-s001.tif]
