# Supplementary material for: Fibroblast growth factor receptor 3 protein is overexpressed in oral and oropharyngeal squamous cell carcinoma
Source: Cancer Med. 2015 Dec 28;5(2):275–84. doi: 10.1002/cam4.595 (PMC4735780; doi:10.1002/cam4.595)
Supplement: Supplementary file 2 — Table S1. FGFR3 protein expression and FGFR3 gene copy‐numbers in oral and oropharyngeal squamous cell carcinoma. [file CAM4-5-275-s002.docx]

| Supplementary Table 1. FGFR3 protein expression and *FGFR3* gene copy-numbers in oral and oropharyngeal squamous cell carcinoma. | | | | | | |
| --- | --- | --- | --- | --- | --- | --- |
|  | OSCC n (%) | OPSCC n (%) | P | OPSCC HPV+ n (%) | OPSCC HPV- n (%) | P |
| FGFR3 protein expression |  |  |  |  |  |  |
| total number of cases | 212 (100) | 240 (100) |  | 43 (100) | 188 (100) |  |
| median (range) | 30 (0-90) | 40 (0-92) |  | 33 (0-92) | 42 (0-90) |  |
| overexpression (>33%) | 89 (42) | 124 (52) | 0.034 | 20 (47) | 97 (52) | 0.489 |
| low expression (<33%) | 96 (45) | 87 (36) |  | 18 (42) | 68 (36) |  |
| missing | 27 (13) | 29 (12) |  | 5 (12) | 23 (12) |  |
| *FGFR3* gene copy-number |  |  |  |  |  |  |
| total number of cases | 212 (100) | 240 (100) |  | 43 (100) | 188 (100) |  |
| amplification | 0 (0) | 0 (0) |  | 0 (0) | 0 (0) |  |
| copy-number gain | 1 (0.5) | 0 (0) |  | 0 (0) | 0 (0) |  |
| normal copy-number | 131 (62) | 192 (80) |  | 36 (84) | 149 (79) |  |
| no signal | 47 (22) | 12 (5) |  | 1 (2) | 10 (5) |  |
| missing | 33 (15.5) | 36 (15) |  | 6 (14) | 29 (16) |  |
| FGFR3 protein expression was determined by immunohistochemistry and *FGFR3* gene copy-numbers was determined by fluorescence *in situ* hybridization on formalin-fixed paraffin-embedded tissues of oral and oropharyngeal squamous cell carcinoma. Nine OPSCC were excluded from the analysis of FGFR3 protein expression in HPV-positive and –negative OPSCC because their HPV status was missing. FGFR3: fibroblast growth factor receptor 3, HPV: human papillomavirus, OPSCC: oropharyngeal squamous cell carcinoma, OSCC: oral squamous cell carcinoma | | | | | | |
